# FEASIBILITY AND EFFECTS OF HIGH-INTENSITY INTERVAL TRAINING ON COGNITIVE FUNCTION AND BRAIN HEALTH IN PERIMENOPAUSE

**DOI:** 10.1093/geroni/igae098.3482

**Published:** 2024-12-31

**Authors:** Victoria Cirone, Nárlon Cássio Boa Sorte Silva, Cindy Barha, Todd Handy, Ryan Stein, Sarah Heath, Teresa Liu-Ambrose

**Affiliations:** University of British Columbia, Vancouver, British Columbia, Canada; University of British Columbia, Vancouver, British Columbia, Canada; University of Calgary, Calgary, Alberta, Canada; University of British Columbia, Vancouver, British Columbia, Canada; University of British Columbia, Vancouver, British Columbia, Canada; University of British Columbia, Vancouver, British Columbia, Canada; University of British Columbia, University of British Columbia, British Columbia, Canada

## Abstract

Neuroendocrine changes during perimenopause negatively impact cognitive function and brain health (e.g., reduced verbal episodic memory and myelin catabolism in the brain). Exercise promotes cognitive performance and mitigates vascular risk factors for cognitive impairment. High-intensity interval training (HIIT) involves short bursts of high-intensity exercise interspersed with active recovery; it improves cardiovascular fitness and vascular risk profile. HIIT addresses the “lack of time” barrier to exercise that perimenopausal females report. This pre-post pilot study assessed the feasibility and potential effects of 12-weeks of HIIT on cardiovascular fitness, verbal episodic memory, hippocampal volume, and myelin content. Feasibility metrics included recruitment rate (i.e., 1/month), withdrawal rate (i.e., < 15%), adherence (i.e., >60%). Training fidelity was assessed by cardiovascular fitness (VO2max.) Verbal episodic memory was assessed by Rey Auditory Verbal Learning Test delayed recall. Hippocampal volume was measured by T1-weighted magnetic resonance imaging. Myelin content was measured by myelin water fraction. Cohen’s d and one-tailed paired t-tests assessed effect sizes and potential changes. Recruitment rate was 0.35 participants/month (six participants in 17 months). Withdrawal rate was 16.67% and adherence was 88.2%. The intervention had a negligible effect on VO2max (Cohen’s d=0.06, p=0.35), large and significant effect on delayed recall (Cohen’s d=0.94, p=0.04), negligible effect on hippocampal volume (Cohen’s d=0.06, p=0.18), and medium to large effect on sagittal stratum myelin content (Cohen’s d=0.68, p=0.06). Future studies should consider home-based exercise and tailored recruitment methods (e.g. social media) to meet recruitment targets. HIIT may be beneficial for cognitive function and myelin content.

